# Patterns of choice adaptation in dynamic risky environments

**DOI:** 10.3758/s13421-021-01244-4

**Published:** 2022-03-08

**Authors:** Emmanouil Konstantinidis, Jason L. Harman, Cleotilde Gonzalez

**Affiliations:** 1 Department of Psychology, University of Warwick, Coventry, UK; 2 Department of Psychology, Louisiana State University, Baton Rouge, LA USA; 3 Dynamic Decision Making Laboratory, Department of Social and Decision Sciences, Carnegie Mellon University, Pittsburgh, PA USA

**Keywords:** Dynamic decision making, Risky choice, Change detection and adaptation, Instance-based learning, Memory processes, Reinforcement learning

## Abstract

An important aspect of making good decisions is the ability to adapt to changes in the values of available choice options, and research suggests that we are poor at changing behavior and adapting our choices successfully. The current paper contributes to clarifying the role of memory on learning and successful adaptation to changing decision environments. We test two aspects of changing decision environments: the direction of change and the type of feedback. The direction of change refers to how options become more or less rewarding compared to other options, over time. Feedback refers to whether full or partial information about decision outcomes is received. Results from behavioral experiments revealed a robust effect of the direction of change: risk that becomes more rewarding over time is harder to detect than risk that becomes less rewarding over time; even with full feedback. We rely on three distinct computational models to interpret the role of memory on learning and adaptation. The distributions of individual model parameters were analyzed in relation to participants’ ability to successfully adapt to the changing conditions of the various decision environments. Consistent across the three models and two distinct data sets, results revealed the importance of *recency* as an individual memory component for choice adaptation. Individuals relying more on recent experiences were more successful at adapting to change, regardless of its direction. We explain the value and limitations of these findings as well as opportunities for future research.

## Introduction

Most choices made at the organizational and personal levels, under risk or uncertainty, are encountered in changing environmental conditions. For example, new technological advancements may increase or reduce the demand for an organization’s products or services. A decade ago, online shopping may have been less attractive and indeed less optimal than visiting a store, given high shipping and other associated costs. This situation has gradually changed: shipping costs have decreased, technology has advanced, and, because of convenience, online shopping has become more attractive compared to shopping at stores, changing the relative value of the alternatives over time. In this, as well as in many other examples, it is important to become aware of the change in the value of the available options to adapt our choices and maximize benefits.

To adapt our choices to changes in naturalistic settings, we often infer the dynamic changes of a situation from the decisions we make and the experienced outcomes, and rely less on descriptions of the pros and cons of each alternative (Gonzalez, 2017; Gonzalez et al., 2017). Experience often takes priority over having a description of the attributes of available choice options, and research suggests that descriptions (e.g., payoffs and associated probabilities) end up being ignored altogether or discounted when making repeated risky choices (Lejarraga & Gonzalez, 2011; Weiss-Cohen et al.,, 2016; but see Weiss-Cohen et al.,, 2018; Weiss-Cohen et al.,, 2021).

However, with heavy reliance on experience, the direction in which available options change over time and the actual individual experiences can influence the way people adapt their choices. In the shopping example above, the experience of online shopping has become more positive over time, e.g., with advancements in technology and easiness of use. An early adopter may have experienced negative outcomes such as long delivery times and product returns, and reconsidered store shopping, while a late adopter may have had more positive experiences with online shopping and been more likely to avoid store shopping in the future.

In this research, we investigate how personal experience, the direction in which available choice options change (i.e., increasing or decreasing in value), and the availability of information (i.e., full or partial feedback about choice options) influence how people adapt their choices in changing decision environments. To shed light on these effects, we turn to a long-standing line of research: experience-based choice between risky monetary gambles (see e.g., Barron & Erev, 2003; Hertwig et al.,, 2004). The *decisions from experience* (henceforth, DfE) literature has accumulated valuable evidence for explaining and predicting choice behavior over time, in situations in which people learn the outcomes and their associated probabilities from feedback received after selecting available choice options (Hertwig & Erev, 2009). In particular, as we will describe below, recent investigations on adaptation to change in experiential choice (e.g., Avrahami et al.,, 2016; Kareev et al.,, 2014; McCormick et al.,, 2020) have shown that people find it hard to detect and adapt to change. Research findings point to *stickiness* and *primacy*, (i.e., over-reliance on initial experiences; similar to the “hot stove” effect, see Denrell and March, 2001) and to *recency* (i.e., over-reliance on most recent experiences), as potential mechanisms involved in the detection of and adaptation to change.

### Memory and adaptation to change in experiential choice tasks

Unlike description-based choice tasks where information about choice alternatives (i.e., outcomes and associated probabilities) is explicitly provided to participants (e.g., a choice between *Option A*: £100 with certainty; and *Option B*: £200 with 50% chance, or 0 otherwise), in experiential choice tasks, participants *learn* (or infer) the value and frequency (i.e., probability) of outcomes from available options by repeatedly choosing among them and observing their outcomes via feedback. The majority of research on risky experiential choice has investigated choice behavior in decision environments where choice options remain unchanged throughout the course of the task (see Erev et al.,, 2010; Hertwig et al.,, 2004; Wulff et al.,, 2018); using the options from the above example in a static experiential choice task, selecting *Option B* would return either £100 or £0 with constant 50-50 probabilities. However, change constitutes a fundamental feature of the real world, and thus exploring behavior in dynamic experiential choice tasks (where outcomes and/or probabilities change from trial to trial) can extend our understanding of decision making in more naturalistic settings (e.g., Navarro et al.,, 2016; Weiss-Cohen et al.,, 2021).

Rakow and Miler (2009) conducted one of the few initial studies that manipulated the way the relative value of choice options changes over time. In a choice task between one dynamic and one static option, the authors investigated how people behaved when static and dynamic options changed their relative expected value during multiple repeated choices. In some environments, the probability of receiving a high outcome from the dynamic option changed from stable to increasing to stable again. This pattern made the dynamic option less favorable than the static option at the early stages of the task, but more favorable later on. Other decision problems involved the reverse pattern of change in the probabilities, from stable to decreasing to stable again, making the dynamic option more favorable than the static option at the early stages of the task and reversing its relative advantage later on. Two interesting, albeit inconclusive, observations emerged from their findings: (1) initial experiences impact later choices and inhibit adaptation to the change in probabilities (i.e., a *stickiness effect*); and (2) individuals seem to respond differently to improvements in a less favorable option than decrements in a more favorable one in the presence of full feedback (i.e., a *direction-of-change* effect). Although stickiness and direction-of-change may be useful to explain the slow adaptation observed in their findings, the authors called for more research to understand how these effects emerge from memory processes and how to design decision environments (i.e., manipulate the direction of change) in ways that adaptation can be predicted.

Recent studies have corroborated and extended the initial results from Rakow and Miler (2009). Avrahami and Kareev (2011) suggested that *recent* rather than earlier experiences are the best and persistent predictors of later choices. They also suggested that inertia (e.g., Dutt & Gonzalez, 2012; Erev & Haruvy, 2005) - a tendency to repeat previous actions (which is a form of *stickiness*) - is partly responsible for the predictive power of recency. It is also claimed that memory is necessary to hold the sequential patterns of observations from a decision environment and to monitor the relative value of options over time (Ashby & Rakow, 2014; Rakow & Miler, 2009). However, some common sequential regularities found in static experience-based choice tasks do not appear to emerge in dynamic tasks with changing probabilities (see Biele et al.,, 2009). Following this, it is reasonable to argue that memory or learning may play a different role in dynamic tasks, and thus, research on their role in such changing environments is necessary.

Theories and models of DfE suggest that choice behavior is determined by well-known aspects of human memory: forgetfulness, confusion of experiences, distortion of the actual probability with which outcomes occur, and over-reliance on experiences from our recent past (Gonzalez et al., 2003; Gonzalez, 2013). In stable environments, higher memory capacity should facilitate the selection of better choice options, because future selections can rely on a larger set of past experiences. Indeed, studies in experiential decision-making have found that higher memory capacity and cognitive resources are good predictors of better performance in static environments (e.g., Fiedler, 2000; Frey et al.,, 2015; Rakow et al.,, 2010). Rakow et al., (2008) observed that the amount of exploration devoted to each alternative (that is, choosing and alternating between options to learn more about their value) was positively correlated with working memory capacity, suggesting that participants would search for more information and are expected to make better choices when they have better memory.

Furthermore, recent research also suggests that memory is important for adaptation to dynamic tasks. Lejarraga et al., (2014) supported Rakow and Miler’s (2009) observations by comparing the predictions from an Instance-Based Learning model (IBL; Lejarraga et al.,, 2012) to observed human choices. Their study compared individual and group adaptation to changing conditions in a dynamic choice task. Using binary-choice experiential tasks similar to those in Rakow & Miler, they found that groups performed better than the average individual in stable conditions of the task, but groups were unable to adapt as well as individuals did after a sudden change in the choice environment. Their explanation was that better memory found in groups can actually be detrimental to adaptation of choices in dynamic conditions. This observation suggests that in dynamic environments, strong memory (i.e., reliance on a large set of past observations) may be unnecessary, because future selections may be best informed by more recent experiences (i.e., small samples; Lejarraga et al.,, 2014; Rakow & Miler, 2009). Studies in DfE have argued that reliance on small samples makes detection of change in the environment easier, and that relying on less information is more advantageous than relying on more samples (Hertwig & Pleskac, 2010). While this intuition seems correct, reliance on recent small samples cannot be entirely attributed to limitations of human memory, but also to the stochasticity of the environment (see Rakow et al.,, 2008).

In the current work, we use a binary choice task to investigate the effects of the direction of change and the type of feedback (full and partial) on participant’s ability to adapt to change. Participants choose repeatedly between a safe option which remains unchanged throughout the task, and a dynamic option which either decreases or increases in expected value across time (through changes in the probability of receiving a high outcome). Participants receive one of two types of feedback: feedback only from the option they select (i.e., partial feedback) or feedback from both the selected and unselected options (i.e., full feedback.

The use of full and partial feedback as an experimental condition is motivated by the limited research on feedback effects in dynamic choice tasks, and by the fact that feedback may be directly linked to the observability of change itself (Avrahami et al., 2016). For example, partial feedback may create a potential asymmetry between the two options and confound the effects of the direction of change. The availability of full feedback suggests that participants will have complete information on the outcomes of both options, while receiving partial feedback will not provide such information unless participants actively select and explore both options. Investigating the direction of change with full and partial feedback would help rule out information asymmetry as a potential confounding factor and test whether any direction-of-change effects are due to environmental situational regularities (e.g., information availability) or genuine characterizations of the decision process (see e.g., Plonsky & Erev, 2017).

To provide a better characterization of the observed behavioral effects and how learning and memory play out in strategies for choice adaptation, we used two distinct computational modeling approaches. The first approach, Instance-Based Learning (IBL), relies on representations of memory mechanisms such as frequency and recency of events to explain decision making in dynamic tasks. Such memory mechanisms, informed by Instance-Based Learning Theory and the well-known cognitive architecture of ACT-R (Anderson & Lebiere, 1998; Gonzalez et al., 2003), explain how decisions from experience depend on memory processes: memory decays over time and the probabilities of events are determined by the frequency of experienced events and are partially stochastic. The IBL model has been used widely in binary choice to replicate human decision making (Gonzalez & Dutt, 2011; Lejarraga et al., 2012).

**Fig. 1:**
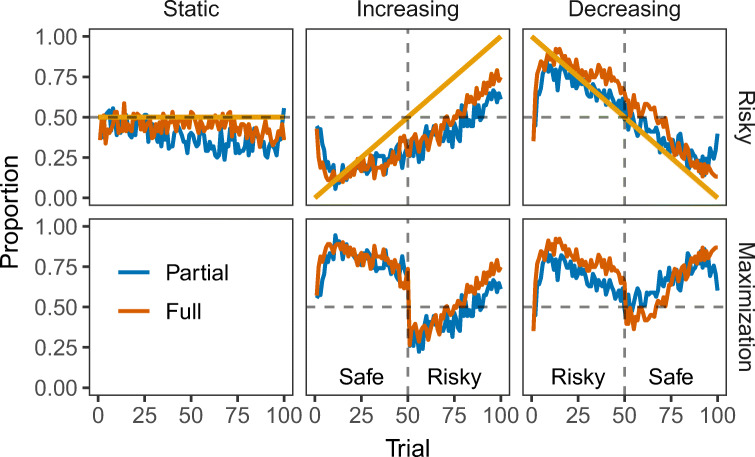
Risky Row: Average proportion of choices from the risky option across trials, direction of change (Static, Increasing, and Decreasing) and feedback presentation (Partial and Full) conditions. Yellow solid lines show the probability of receiving the high outcome (500 points) in the risky option. Maximization Row: Average maximization rates across trials and experimental conditions. Labels in the figure (Safe, Risky) indicate the maximizing option before and after trial 50 (turning point)

The second approach we applied in the current setting is reinforcement learning (RL). Specifically, we used two instantiations (or classes) of model-free RL models (e.g., Sutton & Barto, 1998) which have been extensively used in experience-based decision-making tasks (Yechiam & Busemeyer, 2005). The first instantiation assumes a *delta* rule to update/learn the relative values of each option (see e.g., Busemeyer & Myung, 1992); the second instantiation resembles that of IBL, as it assumes that memory decays as a function of time (hence the values of unchosen options are discounted over time). Crucially, the two modelling approaches assume different learning and choice mechanisms: while RL models assume a trial-by-trial value updating process for each option, the IBL model resembles exemplar-based processes with probabilistic retrieval of the relevant instances/exemplars from memory.

We estimate the parameters of the IBL and RL models at the individual level. This procedure allows us to categorize participants based on their best fitting memory and learning parameters, and relate these parameters to their ability to adapt to the changing environments. The patterns in the individual parameters across two distinct modelling approaches allow us to provide descriptions of the experimental results and make inferences regarding the general role of memory in adaptation to dynamic tasks.

## Experiment: Adaptation to gradual change in a binary choice task

### Participants

We tested a total of 525 participants (*M*_*age*_ = 34.60, *SD*_*age*_ = 11.69; 170 Female), recruited from Amazon Mechanical Turk. They were compensated with both a standard participation fee ($0.50) and an additional payment based on their performance on the task (mean bonus payment = $0.26).

### Task and design

The experimental design was a 2 (Type of Feedback) x 3 (Direction of change) between-subjects experiment. Participants were randomly assigned to one of the 6 conditions to perform a two-option consequential choice task for 100 trials.

The two options were presented in the middle of the screen as two unlabeled buttons set side by side on the right and left of the screen. One option was *safe* and produced a 250 point outcome each time it was selected (i.e., *p* = 1). The second option was *risky* and had two possible payoffs: 0 or 500 points. The probability of receiving the 500 point payoff after selecting the risky option was varied by the direction-of-change condition (Static, Increasing, and Decreasing); and the probability of receiving 0 points for the risky option was the complement. The safe and risky options were randomly assigned to the right or left screen buttons and remained in the same position for the duration of the 100 trials. Also, there were two experimental conditions about whether participants received forgone outcome feedback (Full; feedback about both, the outcome of the selected option and the outcome of the unselected option) or not (Partial; feedback only on the outcome of the selected option).

Figure 1 (straight lines shown in the Risky panel) illustrates the probability of the high outcome (500 points) in the risky option over the course of 100 trials in each of the three direction-of-change conditions. In the Static probability condition (Partial: *N* = 72; Full: *N* = 92), the probability of receiving 500 points from the risky option was .50 throughout the 100 trials. In the Increasing probability condition (Partial: *N* = 54; Full: *N* = 91), the probability of receiving the 500 point outcome began at .01 and increased by .01 on each trial until the final trial where the probability was 1. Finally, in the Decreasing probability condition (Partial: *N* = 78; Full: *N* = 91), the probability of receiving 500 points began at 1 and decreased by .01 on every trial. The two options (safe and risky) in each condition are equivalent in terms of the overall (across all 100 trials) Expected Value (EV): EV(safe)= 250 and EV(risky)= .50 × 500 = 250. However, during the first 50 trials (trials 1 to 49) the risky option is advantageous (i.e., EV maximizing) in the decreasing condition and disadvantageous in the increasing condition. On trial 50 (i.e., the “turning” point), the probability of receiving the high outcome is exactly .50 in all conditions; and following trial 50 (trials 51 to 100) the risky option is disadvantageous in the decreasing condition and advantageous in the increasing condition.

### Procedure

After providing consent and answering demographic questions, participants were given instructions about the task. They were randomly assigned to one of the 6 experimental conditions. The risky and safe options were presented as unlabeled buttons, with their position randomised on the right or left side of the screen. On each trial, participants were asked to select one of the two buttons and were provided with immediate feedback (Partial or Full).

Upon completion of the task, participants filled out a funnel debriefing (see Appendix), designed to ascertain their knowledge of changes in the underlying probabilities of the task. The funnel debriefing asked participants to describe the two choice options, whether they thought one option was better than the other (i.e., it gave the most points on average), and whether they thought one option was better at one point in the game and worse at another point in the game. If participants reported that one option changed, they were then asked whether the target option was better at the beginning or the end of the game, and also asked to indicate on a 0-100 trial sliding scale at which point they thought the option became better (or worse).

Datasets, analysis and modeling scripts are available online on the Open Science Framework (OSF) website: https://osf.io/4swfx/.

## Results

Figure 1 (Risky row) plots the average proportion of choices for the risky option across the 3 (direction of change : Static, Increasing, Decreasing) × 2 (feedback presentation: Partial, Full) between-subjects experimental conditions^1^. Risky rates were analyzed using a generalized logit mixed-effects model with time (blocks of 20 trials), direction of change and feedback presentation as fixed effects, and random intercepts for each participant^2^. The analysis revealed a significant main effect of the direction of change, *χ*^2^(1) = 89.64,*p* < .001, with risky rates being higher in the decreasing (*M* = 0.55) than the increasing condition (*M* = 0.35). The main effect of feedback presentation was also significant, *χ*^2^(1) = 4.98,*p* = .026 (*M*_Partial_ = 0.44, *M*_Full_ = 0.47), but it did not interact with direction of change, *χ*^2^(1) = 1.35,*p* = .25. The main effect of block was significant, *χ*^2^(4) = 51.41,*p* < .001, as well as all 2-way and 3-way interactions with direction of change and feedback presentation (all *ps* < .001). These results indicate that participants were sensitive to the change in the underlying probabilities of the risky option and exhibited distinct choice patterns across experimental conditions.

Specifically, in the decreasing condition, participants took only a few trials before they started to select the risky option at a frequency that appears to match the probability of receiving the higher outcome from the risky option (see top-right panel in Fig. 1A). *Probability matching* (see e.g., Gaissmaier & Schooler, 2008, Shanks et al.,, 2002) has mostly been observed in DfE studies with static probabilities (e.g., Erev & Barron, 2005), but also in dynamic DfE tasks (e.g., Rakow & Miler, 2009). Choice in the increasing condition is not indicative of probability matching. Participants seem to “stick” longer with the safe option even after the turning point (trial 50 onwards), after which selecting the risky option becomes objectively more advantageous. This is the case regardless of whether they received information about the outcome of the unselected option (Full feedback condition).

In the Static condition participants showed an emerging preference for the safe option. However, there were more selections from the risky option in the full feedback group (*M*_Full_ = 0.44 vs *M*_Partial_ = 0.38), which is consistent with findings of increased risk taking in the presence of foregone payoffs (e.g., Erev & Barron, 2005; Weiss-Cohen et al.,, 2021; Yechiam & Busemeyer, 2005).

We performed similar analyses on the maximization rates (Fig. 1, Maximization row) using direction of change, feedback presentation, and time period (two levels; before and after trial 50, the turning point) as fixed effects, and participant-specific random intercepts. The analysis showed a significant main effect of direction of change, *χ*^2^(1) = 6.81,*p* = .009, with participants in the decreasing condition selecting more often the maximizing option than participants in the increasing condition (*M*_Decreasing_ = 0.69 vs *M*_Increasing_ = 0.64). Feedback presentation was also significant, leading to higher maximization rates when full feedback was available (*M*_Full_ = 0.68 vs *M*_Partial_ = 0.64; *χ*^2^(1) = 10.98,*p* < .001), but the interaction with direction of change was not significant, *χ*^2^(1) = 0.02,*p* = .88. Participants maximized more in the first period of the task (before the turning point; *χ*^2^(1) = 1400.49,*p* < .001) and the interaction between direction of change and period was significant, *χ*^2^(1) = 383.20,*p* < .001: Maximization rates in the increasing condition were higher than the decreasing condition before the turning point, but significantly lower after the turning point, suggesting lower levels of adaptation to change in the increasing condition, irrespective of feedback availability (full or partial).

One would assume that the presence of full feedback would allow for equal levels of adaptation in both direction-of-change conditions. Any differences between the two conditions would be found in the partial feedback group and attributed to the information asymmetry caused by the hot-stove effect (Denrell & March, 2001). The effect suggests that early negative experiences have a prolonged effect on choice, leading to fewer selections from the option that generated these negative experiences (or outcomes). In the case of the increasing condition with partial feedback, participants would not experience the change in the relative value of the risky option (i.e., the “hot-stove”, as it mostly returns 0 at the initial stages of the task) because they would under-explore this option in fear of receiving unfavourable 0 outcomes. However, with full feedback, the hot-stove effect is no longer relevant as participants can observe the outcomes of the “hot-stove” without experiencing them: such (foregone) outcomes are not consequential, but provide full information about the outcomes of a choice option. In the increasing condition, early avoidance of the risky option would be reversed by the presence of foregone outcomes, causing participants to switch to the risky option as soon as they realized the change in the probabilities of the dynamic risky option (i.e., more frequent 500 outcomes from the risky option after trial 50). However, this pattern is not observed here; in fact, Fig. 1 shows that the maximization rate in the increasing condition (for both types of feedback) exceeded 50% only after trial 75; at the same time (trial 75), the maximization rate in the decreasing condition was already above 75%.

To examine the impact of any hot-stove effects on choice adaptation, we focused on the significant 3-way interaction (Direction × Feedback × Period). If the maximization rates are similar in the two direction conditions with full feedback, it would suggest that the direction effect is caused by the hot-stove effect (that is, “stickiness” in the increasing condition results from not realizing that the unselected option becomes better across time). We found a significant difference between increasing (*M* = 0.52) and decreasing (*M* = 0.64;OR = 0.60,*z* = − 5.11,*p* < .001) conditions in the second period of the task, suggesting that the hot stove effect is not the sole driver of differences in choice adaptation. The same effect is also observed with partial feedback (*M*_Increasing_ = 0.44 vs *M*_Decreasing_ = 0.67;OR = 0.40,*z* = − 7.67,*p* < .001). These results suggest that the information asymmetry between partial and full feedback cannot account for the direction of change effect in people’s ability to detect and adapt to changes in the environment.

Taken together, the empirical results suggest that slower choice adaptation in the increasing condition cannot be solely attributed to hot-stove effects: in both partial and full feedback groups, we observed that the direction in which the change occurs has a direct effect on choice and adaptation. In the following sections we attempt to shed more light on the determinants of these effects by incorporating a) participants’ perceptions of the change in the environment via the funnel debriefing questionnaire, b) computational modelling analyses), and c) data from a different study that used a similar design but in which both options were risky.

**Fig. 2:**
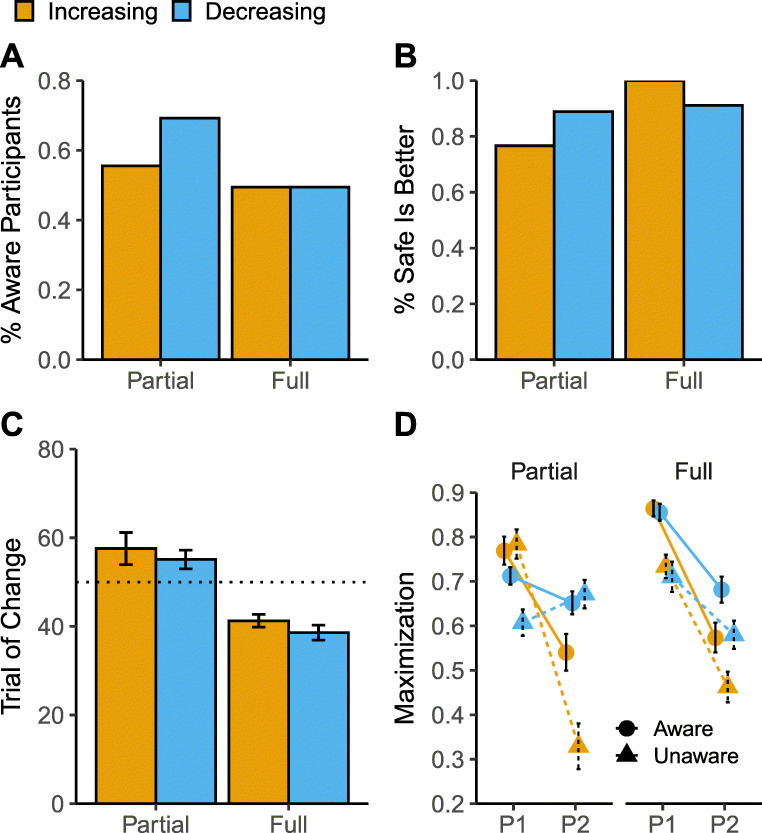
A) Proportion of aware participants across feedback presentation (Partial and Full; x-axis) and direction of change (Increasing and Decreasing). B) Proportion of aware participants that correctly identified the stage (beginning or end of the task) in which the safe option was better than the risky (Q3). C) Mean trial estimate of change (Q4). D) Maximization rates across time periods (P1: trials 0-50 and P2: trials 51-100), feedback presentation, direction of change, and awareness groups (aware and unaware)

### Questionnaire on the awareness of change

To explore participants’ explicit awareness of the change in the probabilities, we classified participants as “aware” (or “unaware”) of the change according to their responses to the questionnaire (see Appendix for explanation of the classification procedure). Fig. 2A shows the proportion of aware participants in each condition. The decreasing condition with partial feedback had the largest proportion of participants aware of the change. Of those participants classified as aware of the change, the vast majority correctly identified the stage in which the safe option was better than the risky option (Q3: Fig. 2B). Furthermore, aware participants were very close at identifying the trial at which the objective change occurred (i.e., trial 50) as shown in Fig. 2C; there is an effect of the availability of full feedback, suggesting that participants were able to realize the change in the environment earlier (around trial 40) compared to aware participants in the partial feedback group who located the start of the change around trial 60.

Explicit awareness of change led to different choice behavior as illustrated in Fig. 2D. The maximization rates across conditions (separately for the two periods, P1 and P2, of the task) were analyzed in a similar fashion as in the main behavioral analysis presented above. Overall, we found that aware participants maximized more than unaware participants, *χ*^2^(1) = 61.26,*p* < .001, and the difference increased in the second period as indicated by a significant interaction, *χ*^2^(1) = 5.18,*p* = .02. This difference was more pronounced in the increasing condition, as indicated by a significant 3-way interaction, *χ*^2^(1) = 50.41,*p* < .001. In other words, becoming aware of the change after the turning point (P2) has a larger effect in the increasing than the decreasing condition. We also found that the 3-way interaction with feedback presentation × period × awareness of change was significant, *χ*^2^(1) = 8.78,*p* = .003, but this is because of a rather large difference between the Partial and Full feedback groups in the aware group before the turning point (P1). Overall, these results suggest that awareness of the changes in the environment leads to advantageous performance in experience-based tasks (see also Konstantinidis & Shanks, 2014; Maia & McClelland, 2004; Newell & Shanks, 2014).

## Computational modelling and choice adaptation

The literature discussed in the introduction suggests a number of explanations for the observed behavioral results. The “stickiness” effect may be an explanation for the slow adaptation in the increasing condition. Initial experiences impact later choices (Rakow & Miler, 2009; Avrahami et al., 2016), as evidenced by the fact that participants in the increasing condition mostly stayed with the safe option even after the turning point. In contrast, faster adaptation in the decreasing condition may be due to strong recency effects (Avrahami et al., 2016), where most recent experiences help participants realize a decrease in the frequency of the best outcome, which causes increased exploration and selection of the safe option. The slight emergent preference for the safe option in the static condition is consistent with diminishing sensitivity and risk aversion in the gains domain, which is consistent with a range of studies in experience-based decision-making (e.g., Erev et al.,, 2008; Ert & Yechiam, 2010; Konstantinidis et al.,, 2018).

Cognitive modeling that incorporates memory and learning/updating processes, can help explain the differences in behavior between the two changing probability conditions, and provide a coherent descriptive account for the behavioural results. For example, whether “good memory” - remembering and tracking changes in past experiences - is an essential component for adaptation; or perhaps just remembering recent experiences can be beneficial for adaptation. Cognitive models of human memory and learning via experience can provide insightful interpretations of the observed behavioral results and a common and integrated theoretical account of the mechanisms likely involved in choice adaptation.

In the next sections, we will first introduce two well-known computational modeling approaches: an Instance-Based Learning model (IBL) and two Reinforcement Learning models (RL). Second, we will use the individual best-fitting parameters to help describe choice and adaptation in each experimental condition. To anticipate our results, the present modeling exercise suggests that memory, as an individual trait, is essential for successful adaptation to changes in the environment. In particular, we find robust evidence for recency, a mechanism necessary for successful adaptation to changes in the dynamic probability task, regardless of the direction of change. We also present a model generalization analysis using the IBL model. This analysis illustrates the predicted adaptation behavior of individuals with high and low recency parameters across different directions of change (e.g., from increasing to decreasing and vice versa). This exercise helps strengthen the conclusions drawn from the model parameters analyses: recency is an individual trait important for adaptation to changing environments, and not a result of the direction of change in the environment. Finally, we show that the results of the present modeling analysis can replicate in a new dataset that uses a similar choice paradigm as in the current study.

### Instance-Based Learning model

The IBL model of binary choice has proved quite successful at capturing choice behavior in both static and changing environments (see Gonzalez & Dutt, 2011; Lejarraga et al.,, 2012; Lejarraga et al.,, 2014). One fundamental assumption in IBL theory (Gonzalez et al., 2003) is that choice occurs by activating memories about past experiences (e.g., observed outcomes) associated with each option/decision. The activation of memory is modulated by at least two processes (i.e., free parameters in the model): memory decay and noise associated with the retrieval of these memories. The activation of outcome *i* in each option *j* on trial *t* is illustrated in the following equation (for a full version of the activation equation in ACT-R including other memory processes, see Anderson & Lebiere, 1998):

1 $$ A_{j,i,t} =\sigma  \ln  \left( \frac{1-\gamma_{j,i,t}}{\gamma_{j,i,t}}\right) + \ln  \sum\limits_{t_{p} \in \{1,...,t-1\}} (t-t_{p})^{-d}  $$

where *d* is a decay parameter, *σ* is a noise parameter, *γ*_*j*,*i*,*t*_ is a random sample from a uniform distribution (between 0 and 1), and *t*_*p*_ denotes all the previous trials that outcome *i* was observed. Lower values of *d* indicate longer-lasting past memories (higher values of *d* suggest strong recency effects). Overall, the first part of the equation is associated with noise in retrieval from memory, whereas the second part is related to the exponential decay of past memories. The activation of each instance in memory determines how likely it is to be retrieved. The probability of retrieval of each instance *i* is relative to the activations of other outcomes observed from choosing option *j*:

2 $$ P_{j,i,t} = \frac{e^{A_{i,t/\tau}}}{{\sum}_{j} e^{A_{j,t}/\tau}}  $$

where *τ* is random noise defined as $\tau =\sigma \sqrt {2}$. Finally, the model chooses the option with the highest *blended* value *V*:

3 $$  V_{j,t} = \sum\limits_{i=1}^{n}P_{i,t}x_{i} $$

where *x* is the value of the observed outcome *i* from option *j*, *P* is the probability of retrieval of this outcome as defined in Equation 2, and *n* is the number of unique outcomes in option *j*. The IBL model accounts for the full feedback condition by creating an additional instance *A* for the outcome *i* produced by the unselected option *j* on trial *t* (that is, each trial *t* is associated with two instances as opposed to one instance in the partial feedback condition; see also Gonzalez et al.,, 2011).

### Reinforcement learning models

Similar to IBL, RL models assume that agents update the value of each choice option based on the observed reward from choosing it; this is instantiated by a learning or updating rule, which keeps track of each option’s expected value. The first RL model is a *delta* learning rule (hereafter, DELTA), which has been extensively used in experience-based decision-making tasks (e.g., Busemeyer & Stout, 2002; Konstantinidis et al.,, 2014; Steingroever et al.,, 2014; Weiss-Cohen et al.,, 2016; Weiss-Cohen et al.,, 2018; Yechiam & Busemeyer, 2005):

4 $$  E_{j,t}= E_{j,t-1} + \delta_{j,t} \phi(r_{j,t} - E_{j,t-1}) $$

According to this rule, the expected value (or expectancy *E*) of an option *j* on trial *t* is the sum of the previous trial *E* and the adjustment based on the prediction error, *r*_*j*,*t*_ − *E*_*j*,*t*− 1_, which is the difference between the reward received on trial *t*, *r*_*j*,*t*_, and the expectancy up to trial *t* − 1. The adjustment is governed by the updating parameter *ϕ* (ranged between 0 and 1); values close to 0 indicate small updating, better memory, and weak recency effects. The delta rule only updates the expectancy *E* of the chosen option, whereas the values of the unchosen options remain unchanged until they are selected; this is coded by the dummy parameter *δ*, which takes a value of 1 if option *j* is chosen, and 0 otherwise. As with the IBL model, we did not apply any modifications to account for the presence of foregone payoffs; the model updates the expectancies of both options as if they were both received (for alternative specifications see e.g., Yechiam & Busemeyer, 2005; Yechiam & Rakow, 2012).

The second RL model contains a decay learning rule (hereafter, DECAY), in which the value of the chosen option is updated, whereas the value of the unchosen options is discounted based on the following decay rule (Erev & Roth, 1998; Speekenbrink & Konstantinidis, 2015; Yechiam & Busemeyer, 2005):

5 $$  E_{j,t}=d_{RL}E_{j,t-1} + \delta_{j,t}r_{j,t} $$

where 0 ≤ *d*_*RL*_ ≤ 1 is the decay parameter: smaller values indicate higher updating and strong recency effects (the dummy variable *δ* has the same meaning as in Equation 4).

Choice in RL models was implemented by a softmax choice rule with a single inverse-temperature parameter *𝜃*, which defines the probability with which each option is chosen on a given trial:

6 $$ P(C(t)=j)=\frac{e^{\theta E_{j,t}}}{{\sum}_{k=1}^{k} e^{\theta E_{k,t}}}  $$

### Model fitting results

We estimated the IBL’s decay, and RL’s DELTA and DECAY parameters by fitting the models to each individual using maximum likelihood estimation (MLE) procedures. Unlike the RL models (DELTA and DECAY), the IBL model used a deterministic choice rule and a stochastic component in the activation function (*γ*_*i*,*t*_ in Equation 1) which is problematic for fitting individual participants using log-likelihood procedures. To overcome these difficulties, we generated 1,000 predictions for each trial and participant under each parameter set *𝜃* (a combination of *d* and *σ*). Thus, the probabilistic model prediction for each participant and trial *t* was the average of these 1000 predictions (see for a similar procedure, Lejarraga et al.,, 2014). We tested all parameter combinations (grid search) for 0 < *d* < 5 and 0 < *σ* < 3 with 0.01 increments. For the RL models, the fitting procedure was a combination of grid-search (100 different starting points for each set of parameters) and Nelder-Mead simplex search methods.

**Fig. 3:**
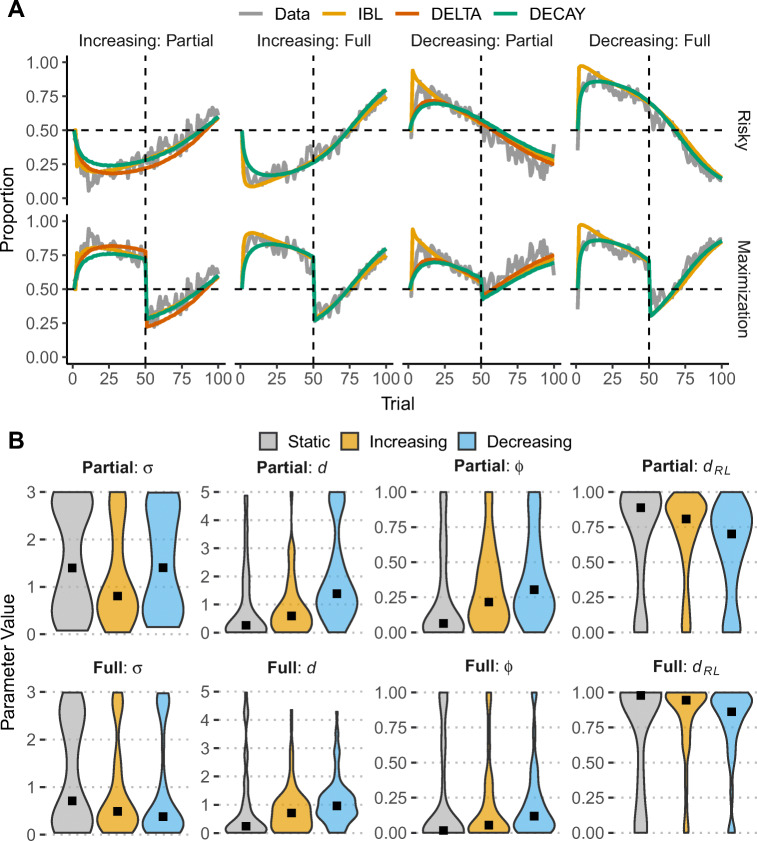
A) Average proportion of risky choice (Risky) and maximization rates (Maximization) for human data (Data: gray line) and the IBL and RL models across trials and experimental conditions (colored lines). B) Distributions (violin-plots) of the best fitting noise *σ* and decay *d* (IBL), and *ϕ* (DELTA) and *d*_*RL*_ (DECAY) parameters across experimental conditions. Square points represent median values

Figure 3A shows model predictions for risky choices and maximization rates for each direction of change and feedback condition^3^. These results suggest that all three models fit the data well and closely track participants’ choices during 100 trials. All models reproduce the main behavioral observations in the dynamic conditions: (1) slow adaptation to the underlying probabilities and suboptimal maximization in the increasing condition, regardless of feedback (partial or full); (2) faster adaptation, probability matching, and higher maximization in the decreasing condition.

To explore whether successful adaptation to change can be explained based on the memory and learning mechanisms assumed in the models, we investigated the distributions of individual model parameters. Figure 3B shows the distributions (violin-plots) of model parameters across direction of change and feedback conditions. The figure also includes the Static condition as a comparison group. A first inspection of the differences between parameters provides explanations for the observed choice patterns in the direction of change conditions.

Specifically, in the partial feedback conditions (first row in Fig. 3), the IBL decay parameter *d* is higher in the decreasing than the increasing and static conditions (*Mdn*_Decreasing_ = 1.39, *Mdn*_Increasing_ = 0.60, *Mdn*_Static_ = 0.26; Kruskal-Wallis Test: *H*(2) = 36.03,*p* < .001), which suggests that participants in the decreasing condition relied more on recent information, resulting in higher maximization in the second period of the task (after trial 50; see Fig. 3A). There were no significant differences between conditions regarding the IBL noise *σ* parameter, *H*(2) = 2.90,*p* = .23.

The RL learning/updating and decay parameters show similar patterns: Higher values of the DELTA *ϕ* parameter (*Mdn*_Decreasing_ = 0.30, *Mdn*_Increasing_ = 0.22, *Mdn*_Static_ = 0.06; *H*(2) = 18.24,*p* < .001) and smaller values of the DECAY *d*_*RL*_ parameter (*Mdn*_Decreasing_ = 0.70, *Mdn*_Increasing_ = 0.81, *Mdn*_Static_ = 0.89; *H*(2) = 13.18,*p* = .001) in the decreasing condition. These results, obtained independently from different models, suggest higher memory updating, higher decay, better adaptation, and less “stickiness” to the previously learned values of each option in the decreasing condition. The learning/decay parameters also suggest that participants show a greater degree of “stickiness” to the previously good option in the increasing condition (see also Rakow & Miler, 2009).

Similar patterns about differences between the changing probability conditions are observed in the full-feedback conditions (second row in Fig. 3): participants in the decreasing condition were characterized by higher decay *d* (*Mdn*_Decreasing_ = 0.96, *Mdn*_Increasing_ = 0.71, *Mdn*_Static_ = 0.24; *H*(2) = 23.25,*p* < .001), higher *ϕ* updating (*Mdn*_Decreasing_ = 0.12, *Mdn*_Increasing_ = 0.06, *Mdn*_Static_ = 0.02; *H*(2) = 11.62,*p* = .003), and smaller *d*_*RL*_ (*Mdn*_Decreasing_ = 0.86, *Mdn*_Increasing_ = 0.94, *Mdn*_Static_ = 0.98; *H*(2) = 7.71,*p* = .02). We also observed that across changing probability conditions all three updating/decay parameters (*d*, *ϕ*, and *d*_*RL*_) have higher values in the partial-feedback than in the full-feedback conditions. Similarly, differences between changing probability conditions are more pronounced in partial-feedback. Also, the parameter values in the static condition for both partial and full feedback provide further support for the close link between recency and learning processes and adaptation to change: as the choice environment in the static condition stayed invariant during the course of the task, all three updating/decay parameters (*d*, *ϕ*, and *d*_*RL*_) showed reduced memory updating or decay compared to the dynamic conditions.

**Fig. 4:**
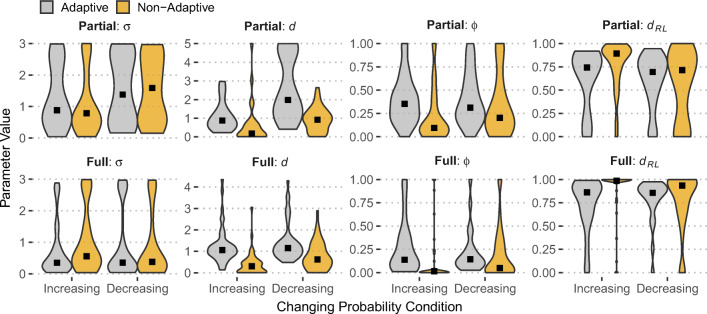
Distributions of model parameters (violin plots) for adaptive and non-adaptive participants across changing probability (Increasing; Decreasing) and feedback presentation conditions (Partial and Full). Black square points represent median values

### Memory and recency in adaptive and non-adaptive participants

To analyze how the models explain adaptive behavior, we categorized participants in those who were adapted to change (“Adaptive”) and those who did not (“Non-Adaptive”). This categorization was the result of a median split on maximization performance in the last 30 trials of the task (trials 71-100), which provides enough time to notice the objective change that happens at trial 50. The main question is whether successful adaptation to change can be explained based on memory and learning mechanisms in dynamic environments, as assessed by the parameters of the three models used here.

Figure 4 shows model parameter distributions separately for Adaptive and Non-Adaptive participants across changing probability and feedback conditions. The pattern that stands out is that all three learning/decay parameters (*d*, *ϕ*, and *d*_*RL*_) show distinct parameter profiles between the two adaptation groups in both Partial and Full feedback, indicating that successful adaptation to change and higher maximizing rates are captured (in the models used here) by higher decay (IBL and DECAY models) and higher learning rates (DELTA model). This effect is suggestive of the ability of these models to discriminate between different choice profiles and degrees of adaptation to change.

### IBL model generalization

The analysis in this section aims to illustrate how individuals characterized by high or low recency would adapt to different conditions of direction of change (e.g., from increasing to decreasing conditions and vice versa). This exercise helps explore whether model parameters can only be considered in the decision environment in which they have been calibrated/fitted, or whether they represent unique psychological features of each individual (see related discussion in Ballard et al.,, 2021; Gonzalez & Dutt, 2011; Harman et al.,, 2021).

One issue of interest is whether individuals characterized by high recency in the decreasing condition would maximize equally well in the increasing condition, compared to individuals with low recency. The expectation is that, if high recency is relevant to adaptation, then individuals with high recency would not only show high levels of maximization in the decreasing but also in the increasing condition. Similarly, individuals with low recency in the decreasing condition should be unable to reach high levels of maximization in the increasing condition.

To explore this analysis, we used participants’ best-fitting IBL parameters from a specific decision environment (i.e., *calibration* decision environment) to simulate choice behavior in new experimental conditions (i.e., *test* decision environment). This is similar to “the generalization criterion” discussed in the model comparison literature (Ahn et al., 2008; Busemeyer & Wang, 2000). We split participants into two groups (median split; High recency and Low recency) using their best fitting *d* parameter for each of the dynamic conditions (Increasing and Decreasing) and for each of the feedback groups (Partial and Full). This created 8 groups of combinations of decay *d* parameter, i.e., *High Recency-Increasing*; *High Recency-Decreasing*; *Low Recency-Increasing*; and *Low Recency-Decreasing*, separately for the partial and full feedback groups. For each *calibration* group, we sampled 1,000 random combinations of best fitting parameters (pairs of *d* and *σ* IBL parameters) and simulated behavior using the characteristics (payoffs and probabilities) of the test environment.

**Fig. 5:**
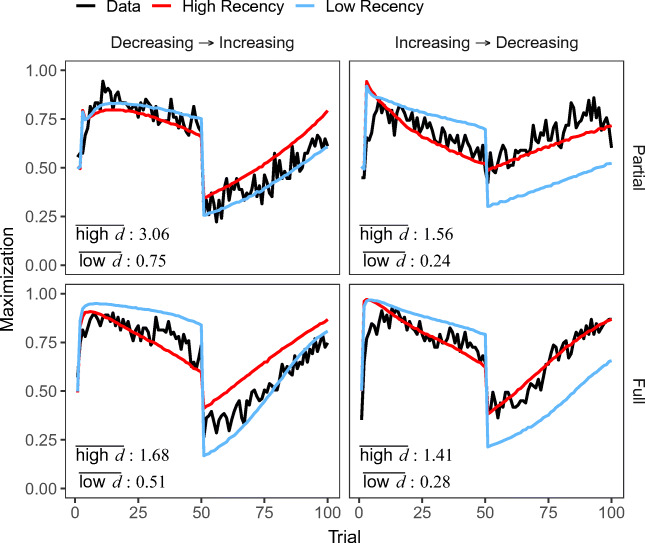
Generalization simulations for partial and full feedback groups using the best fitting parameters from one decision environment (*calibration* environment) to simulate behavior in the other decision environment (*test* environment): *calibration* set → *test* set. Colored lines represent high and low recency, which are plotted against behavior in the test set (black line). Each panel shows the average high and low IBL *d* parameter values, which were estimated in the calibration environment and used to generate behavior in the test environment

The aggregate results of the simulation exercise are presented in Fig. 5. The figure presents simulated behavior in the test condition with parameters fitted (i.e., calibrated) in the calibration condition: “Calibration Condition → Test Condition”. The left panels of Fig. 5 (“Decreasing → Increasing”) show simulated behavior in the Increasing condition, of participants calibrated in the Decreasing condition and classified as high and low recency agents. These results are presented together with the human data observed in the Increasing condition. Similarly, the right panels of Fig. 5 (“Increasing → Decreasing”) show simulated behavior in the Decreasing condition, of participants calibrated in the Increasing condition and classified as high and low recency agents (along with observed human data in the Decreasing condition). It is important to note that the 8 groups of “High Recency” and “Low Recency” are characterized by different average recency (or IBL *d*) values; the average high and low *d* values ($\overline {\text {high} \ d}$ and $\overline {\text {low} \ d}$) used to simulate behavior are shown in each of the four panels in Fig. 5.

It is easy to observe that regardless of the condition in which they were calibrated, simulated agents with high recency maximize more than those with low recency in the second period of the task (trials 51-100). Agents with low recency seem to maximize more with full feedback in the first period of the task (trials 1-50), but they adapt to change in the environment less successfully than agents with high recency. These results suggest that recency is an individual trait, and that individuals with high recency (those that mostly rely on recent experiences) are able to adapt to change better than those with low recency, regardless of the direction of change^4^.

## Choice adaptation and risk preferences

A potential moderating factor of how people adapt to change is individual risk preferences and the composition of the decision environment. Given that our experimental task involved a safe and a risky option, a possible explanation for the observed adaptation patterns in the two directions of change conditions is participants’ willingness to take risks. This is particularly relevant in the context of the present study as the risk is experienced, and these experiences may be unique for each participant. To determine whether the effects of the direction of change and feedback on adaptation are the result of risk preferences, we used the data from McCormick et al., (2020), collected in a study with an identical experimental design to the one reported here, except that in their study a choice was made between two risky options (as opposed to safe vs risky in our study). Specifically, participants made a choice between two risky options that returned the same two outcomes, 0 or 500 points. One option was stationary and returned these outcomes with constant probabilities (*p* = 0.50 for each), whereas the dynamic conditions (increasing and decreasing) were identical to our study.

**Fig. 6:**
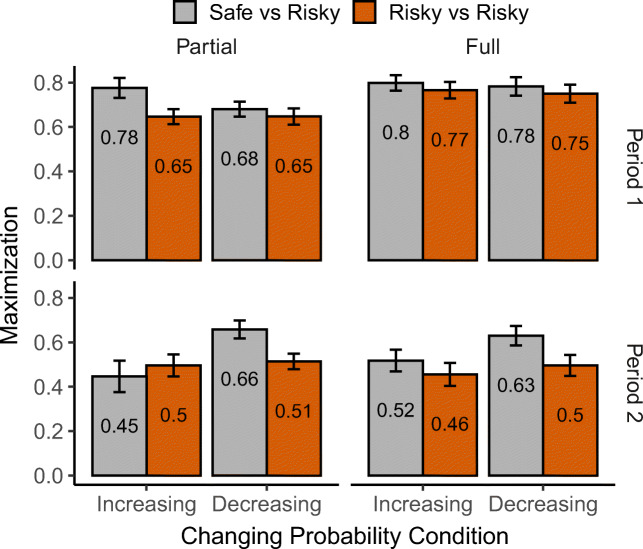
Average proportion of maximization rates for the two studies (Safe-Risky: Present Experiment; Risky-Risky: Study 1 in McCormick et al., 2020) across changing probability conditions (Increasing; Decreasing), feedback presentation (Partial and Full), and task period (Period 1: Trials 1-50; Period 2: Trials 51-100)

We looked at adaptation rates (i.e., maximization behavior in Period 2) in the two task designs (Safe vs Risky: *SR* and Risky vs Risky: *RR*) across direction of change and feedback conditions (see Fig. 6). We analyzed the data as previously with the addition of *design* as a fixed-effect. Overall, there was a 4-way significant Direction × Feedback × Period × Design interaction, *χ*^2^(1) = 57.07,*p* < .001. Following up on the interaction effect, the main contrast of interest is the difference between the direction of change conditions in the RR design. Unlike what we observed in the SR design (i.e., significant direction of change effects in both partial and full feedback groups), in the RR design there are no differences between increasing and decreasing conditions across both feedback groups (both *ps* > 0.083). This largely suggests that participants’ risk preferences and the composition of the choice environment affect choice adaptation in dynamic environments; in other words, the direction of change effect that is present when a safe option is pitted against a risky option seems to disappear when both options are risky.

Combining the results from both designs/studies allows for a clearer interpretation of the observed behavioral effects. First, the effect of the direction of change seems to be immune to the availability of full feedback, but disappears in a choice environment where both options are risky (and provide identical outcomes). This can be further explained by risk aversion in the gain domain, which may have caused the higher maximization levels in the decreasing condition, where the advantageous/adaptive behavior is to select the safe option after the turning point. On the other hand, in the increasing condition, overcoming risk aversion and switching to the risky option possibly requires substantially more evidence (i.e., more frequent high outcomes from the risky option). This is possibly why the effect of the direction of change disappears in the RR design where participants have to choose between risky options.

**Fig. 7:**
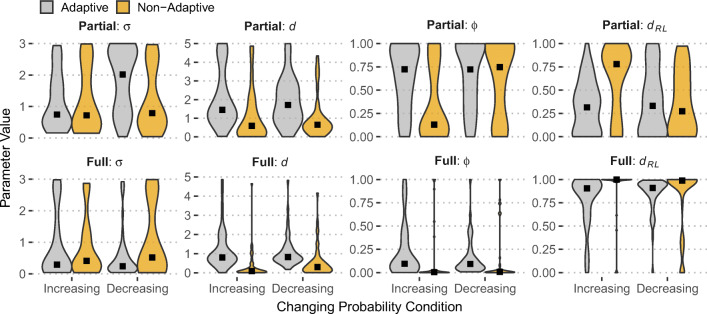
Distributions of model parameters (violin plots) for adaptive and non-adaptive participants across changing probability (Increasing; Decreasing) and feedback presentation conditions (Partial and Full) in Study 1 in McCormick et al., 2020. Black square points represent median values

Despite the moderating effects of individual risk preferences and the environment composition, the question is whether adaptive participants in the RR design would show distinct recency and updating profiles compared to non-adaptive participants. As before, we examined the distributions of model parameters across the three models fitted to individual data in the RR dataset (McCormick et al., 2020)^5^. These results are shown in Fig. 7: There is a clear difference in the IBL *d* parameter between adaptive and non-adaptive individuals in both dynamic conditions and feedback presentation conditions (Partial and Full). In agreement with the results in Fig. 4, adaptive participants are characterized by higher *d*, suggesting more reliance to recent experiences and thus higher likelihood to observe the change and adapt their choices accordingly. Similarly, the RL parameters (*ϕ* and *d*_*RL*_) show differences between the two dynamic conditions: higher *ϕ* and smaller *d*_*RL*_ for adaptive than non-adaptive participants, apart from the decreasing condition in the partial feedback condition.

## Discussion

The main objective of the current work was to provide a characterization of how individuals adapt to changing conditions when they make decisions under risk and uncertainty. This work builds on recent attempts aimed at understanding how people detect and adapt to change when making decisions from experience (e.g., Avrahami et al.,, 2016; Kareev et al.,, 2014; Speekenbrink and Konstantinidis, 2015; Rakow & Miler, 2009; McCormick et al.,, 2020). In addition to contributing new empirical evidence regarding the conditions under which individuals detect and successfully adapt (or not) to change, we also provide potential explanations and descriptions of the observed empirical effects using computational cognitive models.

### Adaptation to change: Behavioral effects

The behavioral effects of the current and related studies (see McCormick et al.,, 2020) can be summarized as follows. First, there are robust differential effects of the direction of change in people’s ability to adapt to change: in the context of our experiment, risk which becomes more rewarding over time (increasing condition) is harder to detect (or harder to accept), compared to risk that becomes less rewarding over time (decreasing condition). This indicates that adaptation to increasing positive outcomes may be adversely affected by initial disappointments (frequent 0 outcomes) which become difficult to correct later. This is consistent with Rakow & Miler’s (2009) observation that people may react and adapt more quickly to negative than to positive changes (see similar observations in Lejarraga et al.,, 2014). In addition, participants’ choices in the decreasing condition show a clear pattern of probability matching behavior: monitoring and experiencing the change in the risky option makes behavior match the change in the probabilities. In addition, the results from the funnel debriefing largely support the observed choice patterns and conclusions: participants who expressed more awareness of the change maximized more than those that were less aware of the change.

Second, the critical direction of change effect cannot be solely attributed to information asymmetry and hot-stove effects. While our initial hypothesis suggested that adaptation difficulties in the increasing condition may stem from information asymmetries between partial and full feedback (and as a result, the hot-stove effect causing under-exploration of the risky option and reduced rates of adaptation), the results showed that, even with full feedback, choice adaptation in the increasing condition lagged behind the decreasing condition. Foregone payoffs helped participants in the increasing condition maximize more compared to the partial feedback group, but this increase did not reach the maximization and adaptation rates in the decreasing condition.

The questionnaire results are also illuminating: while the percentage of participants classified as aware of the change is similar in both direction-of-change and feedback conditions (Fig. 2A), and while these (aware) participants accurately respond to the funnel debriefing questions (Fig. 2B and C), this knowledge does not translate into equal levels of adaptation in the second period of the task for both increasing and decreasing conditions. Figure 2D shows that aware participants in the decreasing condition (for both types of feedback) maximize more than aware participants in the increasing condition (circle markers). Hence, the questionnaire results rule out the possibility that differences in adaptation can be attributed to difficulty or inability to detect that something is changing in the task (which is also supported by the pattern of results in the full feedback condition).

The immunity of the direction-of-change effect to feedback availability and the observation that this does not result from participants being unaware of the change is challenging to existing theories and empirical observations in the DfE literature; future research should explore the conditions under which the effect is observed and the factors that moderate it.

One of these factors may be the composition of the decision environment and individual risk preferences; the data from a study which explored the same instantiation of direction of change (positive linear and negative linear), but with choices between two risky options (RR design; as opposed to choices between one safe and one risky option, SR design) revealed that the effect disappears and differences in adaptation between increasing and decreasing conditions are no longer credible. A potential explanation is risk aversion: participants in the SR design were willing to stay longer with the safe option regardless of the higher expected returns from the risky option in the second period of the task. Another possibility is that both choice options produce identical outcomes (0 or 500) which potentially makes discriminating and choosing between options harder than when options produce different outcomes. Future research can investigate the validity of this hypothesis and, more generally, the aspects of the decision environment that are more predictive of successful and faster adaptation in dynamic conditions. The fact that the task/environment composition can alter or even reverse observed empirical phenomena in experience-based risky choice is not surprising; for example, it has been observed that underweighting of rare events (see Barron & Erev, 2003; Hertwig et al.,, 2004) - a pivotal effect in DfE tasks - may be hard to generalize to choices between two-outcome gambles (see e.g., Glöckner et al.,, 2016).

### Computational modelling and adaptation to change

The question we address with computational cognitive models is whether a common psychological mechanism can explain the observed choice patterns of adaptation. To that end, we used two modeling approaches with a long history in DfE and learning tasks, which also make different assumptions about learning and choice behavior in similar tasks: a cognitive memory-based model (IBL) and two instantiations of RL models. All three models captured choice behavior, replicating the behavioral observations across two different data sets (see Figs. 3 and 7). One robust explanation emerges from the interpretation of the parameter distributions across experimental conditions and datasets: *recency* is directly associated to adaptation to change. Higher decay in IBL and DECAY models and higher learning rates in the DELTA model (and thus higher reliance on recent experiences) explain why participants were able to adapt and adjust their choices faster in the decreasing than the increasing condition. On the other hand, lower decay and updating rates (i.e., more reliance on old experiences, or *primacy*) in the increasing condition explain participants’ prolonged “stickiness” with the safe option. These results highlight the importance of sensitivity to recent events and how reliance on the most recent history (and forgoing older experiences) can be successful at adapting to change in dynamic environments. This recency effect is robust across feedback conditions (partial and full feedback).

Furthermore, we provide novel predictions of how patterns observed with one set of parameters in one environment can generalize into a new environment. The predictions made by the IBL generalization exercise (see Fig. 5) suggest a major conclusion: higher decay and reliance on recent experiences is an essential component for adaptation to change under uncertainty. High decay individuals are able to adapt to the changes in both dynamic conditions (increasing and decreasing) better than low decay individuals. That is, our ability to forgo initial experiences and rely only on more recent experiences results in better adaptation to change; in addition, low decay explains the stickiness to the initially good option and the inability to adapt to the change after the turning point. Thus, regardless of the direction of change, the individual ability to efficiently and strategically “forget” (i.e., individuals with high decay) is essential for adapting to changing environments.

### Limitations and new perspectives on detection and adaptation to change

The study of dynamic experience-based decision-making has many practical implications in real life and an extensive investigation of how people perform in similar tasks will prove beneficial. Compared to static experiential choice environments, the area of detection and adaptation to change in dynamic settings remains largely unexplored. Our present work provides useful behavioral observations regarding adaptation to change: One critical mechanism that is responsible for successful adaptation is the ability to ignore earlier experiences and focus on more recent ones.

We acknowledge, however, the limitations of this initial investigation and the potential boundaries of the explanations found in this research. First, change in the decision environments can be manifested in many ways (Speekenbrink & Shanks, 2010; Gonzalez et al., 2003; Gonzalez et al., 2017; McCormick et al., 2020). In this paper, we explored a particular set of monotonic linear functions of change of the probabilities of the highest outcome. Arguably, this is perhaps not the most common form of change in the world. Indeed, many dynamic systems are not linear and some show periodic changes (e.g., demand of beer in the summer increases while it decreases in the winter Gonzalez et al.,, 2017; Cronin et al.,, 2009). For example, one can expect that in periodic changes, remembering early experiences can be advantageous to adapt to the cyclic conditions of change. Future research in DfE should explore different functional forms of change and behavioral choice patterns - for example, it has been found that linear functions similar to the ones employed in the current work are easier to learn (e.g., DeLosh et al.,, 1997; Schulz et al.,, 2018) and people have shown a bias towards linear functions with positive slope (e.g., see Kalish et al.,, 2007).

Similarly, it would be interesting to examine whether people have explicit knowledge of how probability changes across time. In the current work, we assessed the degree to which participants realized that something was changing in the environment, but future research could try to ascertain whether specific instances of the generating function of change can be recognized, and in turn how they can be used to inform choice dynamics. Also, it is important to note that the objective distinction between static and dynamic settings in DfE tasks may not be perceived as such by participants. For example, a large body of work has shown that even in static tasks people behave as if the environment is dynamic (see Navarro et al.,, 2016; Plonsky et al.,, 2015; Szollosi et al.,, 2019). Thus, understanding participants’ perceptions of the degree of change or volatility in the environment may offer better insights and predictions about choice behavior.

Naturally, it would be important to explore how decision models account for people’s integration of experiences across various functional forms of change. Are the same representations assumed in the IBL and RL models able to account for behavior in different functional forms of change? What are the limits of the human ability to detect changing patterns and act accordingly? These are important questions to address in future research.

## Appendix: : Funnel debriefing questionnaire

Questions 1 and 2 (Q1 and Q2; see below) were designed to assess whether participants in the changing probability conditions realized that the environment was gradually changing. Q1 asked participants to indicate which option they thought was the best throughout the task (i.e., produced the most points overall); they could also respond that *neither* option was better than the other (if participants responded “neither” they were presented with Q2b, otherwise they were presented with Q2a). Q2a asked participants whether they thought that the option they picked in Q1 was the best option at each point during the game. Participants were classified as aware of the change if their response to Q2a was negative or if their response to Q2b was positive. Aware participants were subsequently presented with the remaining questions (Q3 and Q4), which were designed to probe additional features of their knowledge and detection of change.

### Questionnaire:

**Q1**:

Overall, which option do you think was the best (gave you the most points on average)? Possible answers: *A, B, or Neither*

**Q2a** (if A or B is selected in Q1): Do you think option {option selected in Q1} was the best option at each point during the game? Possible answers: *YES or NO*

**Q2b** (if Neither is selected in Q1): Do you think that at specific points in the game one option was better than the other? Possible answers: *YES or NO*

**Q3** (only participants who responded in Q2a = NO, or in Q2b = YES): Do you think option A {the safe option} was better: Possible answers: *Towards the beginning of the game — Towards the end of the game*

**Q4**: Below is a slider that represents the 100 trials of the game. As best as you can, please indicate around which trial option A became better (or worse) than option B.

## Footnotes

1 We excluded 47 participants from further analyses as they chose one option more than 95 out of the 100 trials, which is an indication of extreme lack of exploration (and motivation) to allow for the detection of the change in the choice options.

2 The analysis did not include the Static condition as the main focus of the present work is the changing probability conditions.

3 The model predictions are generated based on simulating 1000 agents from each participant’s best-fitting parameter values.

4 The IBL model predicts that agents with *identical* *d* and *σ* parameter values will have similar levels of maximization and adaptation under conditions of full feedback, regardless of the direction of change (increasing or decreasing). The higher maximization rates in the full feedback group of the decreasing condition (see Fig. 5–Full) are explained by higher average *d* values than the increasing condition ($\overline {\text {high} \ d}$: 1.68 vs 1.41 and $\overline {\text {low} \ d}$: 0.51 vs 0.28).

5 The same IBL and RL models were fitted using the same procedure as the one outlined in the Model Fitting Methods section
